# Electromagnetically Induced Transparency in Media with Rydberg Excitons 2: Cross-Kerr Modulation

**DOI:** 10.3390/e22020160

**Published:** 2020-01-30

**Authors:** David Ziemkiewicz, Sylwia Zielińska-Raczyńska

**Affiliations:** Institute of Mathematics and Physics, UTP University of Science and Technology, Al. Prof. S. Kaliskiego 7, 85-789 Bydgoszcz, Poland; sziel@utp.edu.pl

**Keywords:** electromagnetically induced transparency, cross-Kerr nonlinearity

## Abstract

By mapping photons into the sample of cuprous oxide with Rydberg excitons, it is possible to obtain a significant optical phase shift due to third-order cross-Kerr nonlinearities realized under the conditions of electromagnetically induced transparency. The optimum conditions for observation of the phase shift over π in Rydberg excitons media are examined. A discussion of the application of the cross-phase modulations in the field of all-optical quantum information processing in solid-state systems is presented.

## 1. Introduction

The recent development of various light manipulation techniques arising from electromagnetically induced transparency (EIT) has made it possible to slow down the pulse (photons), store, and retrieve them, preserving the phase relations [1]. At the level of single photons, the EIT enables the quantum information carried by photons to be mapped in the form of quantum coherences inside the medium, effectively creating a quantum memory. It allows one to transfer quantum states between photons and matter. Recently, it has been shown that EIT significantly enhances the optical nonlinearity [2]. EIT has been proposed as a way to greatly enhance cross-phase modulation (XPM), which refers to the phenomenon where the phase of one photon is modulated by another photon [3]. One of the widely explored schemes to enhance cross-phase modulation is based on the Kerr-EIT-like interaction between two weak optical fields [4]. Recently, Bai et al. [5] considered strong Rydberg–Rydberg interactions which are the source of third- and even fifth-order Kerr nonlinearities. Interactions between photons realized by nonlinear optical mechanisms are essential to quantum information processing, quantum teleportation, and quantum logic gates [6,7,8]. Due to the large nonlinear susceptibilities at low light levels, the EIT-based XPM in atomic vapors makes single-photon operations feasible and can lead to applications in quantum information manipulation. XPM has been considered as a promising means of quantum communication and quantum computation. The large nonlinearity at the single-photon level could pave the way for the implementation of universal quantum gates. However, realizing large nonlinearity at such low light levels has been a great challenge for scientists in the past decade [9,10].

Solid bulk media are systems well worth considering for storing and processing quantum information because they have a number advantages over atomic gases, where many experiments have been done (for a recent review see Ref. [11]). They are easy to prepare, diffusion processes are not very fast, and much higher densities of interacting particles can be achieved [12]. One common class of solids used within the quantum information context are the rare-earth-metal-doped crystals, where a long time period of information storage has been achieved (i.e., over one minute) [13,14] Nitrogen-vacancy centers in diamond are also of interest [15], which have a relatively long spin coherence. Another class of solid-state systems where EIT occurs are so-called artificial atoms [16,17,18,19]. Recently, the Rydberg excitons (REs) have attracted a great deal of attention due to their exciting features: the distinct combination of their long radiative life-times, sensitivity to external fields, and strong dipolar interactions [20] could be exploited to realize quantum interfaces for quantum information processing. Rydberg excitonic samples smaller than other solid-state systems mentioned above. The observation of dipolar blockade in bulk Cu_2_O [21], quantum coherence [22], and single-photon source based on RE blockade [23] were performed in samples of micrometer scale. The realization of these experiments has unlocked a plethora of dynamic effects which might be observed in Rydberg excitons media [24,25,26,27]. One example is the electromagnetically induced transparency discussed in our previous paper [28], the performance of which in Cu_2_O bulk crystal will be the next step towards the potential implementation of this medium for quantum information processing. This paper follows up our previous work [28], where the optimal conditions for performing EIT in the linear regime were discussed. Here, by expanding these considerations and results, we propose to explore the scheme that enables one to induce a substantial nonlinear interaction and cross-phase modulation between two slow-light narrow pulses, realized in a cuprous oxide crystal with RE. This nonlinearity may be reached by disturbing the two-photon resonance condition in a two-ladder configuration while keeping the absorption negligible. Our simulations demonstrate the feasibility of achieving large cross-phase modulation in the system with small absorption.

Because slow light experiments can be performed under Autler–Townes or EIT conditions [29,30,31], which are often confused, it should be stressed that in this paper we discuss the case of narrow band operations, for which EIT is the most suitable [32].

Furthermore, we present an overview of the impact of parameters (excitonic states, control field intensities, temperature, or sample size) and provide a realistic example of a system to facilitate an experimental demonstration of XPM for RE in Cu_2_O. The proposed scheme could possibly be used to implement photon–photon quantum gates, demonstrating the potential of Rydberg excitons media as a platform for quantum communications and quantum networking.

Our paper is organized as follows. In Section 2 we outline the theory of cross-phase modulation in an inverted Y system. Then, in Section 3 the results of calculations for a chosen excitonic state combination are presented and the impacts of various conditions on the system are discussed.

## 2. Theory

**Figure 1:**
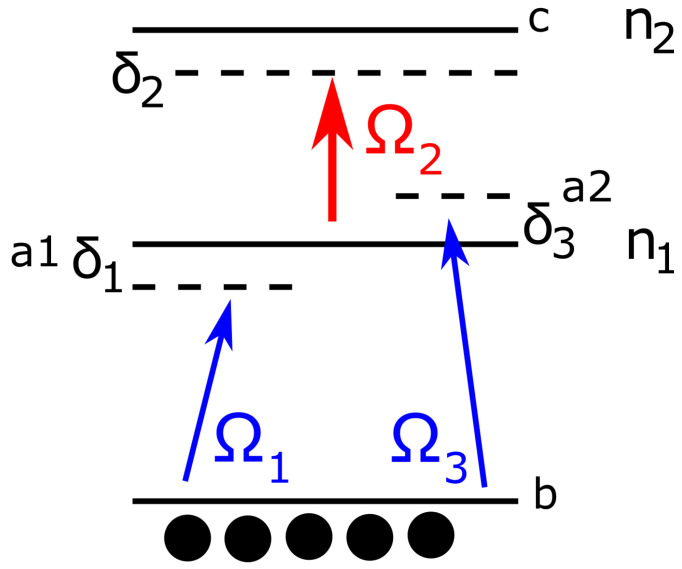
Schematic of the considered inverted Y configuration.

In the following, we consider a Cu_2_O crystal as a medium with Rydberg excitons, where the XPM under conditions imposed by EIT in the linear regime can be realized. We use the so-called inverted Y configuration, which consists of two subsystems of ladder configuration (Figure 1). The whole system is composed of four levels $a_{1},a_{2},b$, and *c*. As in our previous works on Rydberg excitons [28], we focus our attention on the yellow excitonic series. We chose the valence band as the *b* state. As a practical example, the $n_{1}P$ and $n_{2}S$ excitonic states and dipole-allowed transitions WEre chosen. The description of the optically allowed transitions in this system is as follows: the ground state *b*, which is identified with the valence band, is coupled by two weak probe and signal beams of Rabi frequencies $\Omega_{1}$ and $\Omega_{3}$ with states $a_{1}$ and $a_{2}$. These two states are the sublevels of a state *a* obtained by applying a constant external magnetic field producing Zeeman splitting of the *P*-exciton levels [33], which in our case applies only to the *a* ($n_{1}P$) excitonic state. Note that these two weak beams are slightly detuned from the a−b resonance. The two empty upper states *a* and *c* are coupled by the control field of Rabi frequency $\Omega_{2}$. If one of the weak (signal or probe) fields is missing, the system reduces to a standard ladder-type three-level EIT configuration (in the linear case), driven by the control field, which has been considered in our previous paper [28]. With the three fields shown in Figure 1, our scheme acts as a double EIT system with two independent probe and signal fields propagating in the two transparency windows sharing the common control field. In such a situation, we deal with the case of multi-channel propagation under EIT conditions, so two different weak light pulses centered at two independent transparency frequencies travel with slow group velocities through the RE medium.

In order to study the propagation and interaction of the signal and probe fields inside the medium, the set Bloch equations of the form similar to that considered in our previous paper [28] (in the stationary case for the linear EIT regime) is accompanied by the Maxwell propagation equations for the Rabi frequency $\Omega_{1}$ and $\Omega_{3}$ of the probe and signal pulses, which in the slowly varying envelope approximation read

(1) $$\left(\frac{\partial }{\partial t}+c\frac{\partial }{\partial z}\right)\Omega_{1,3}\left(z,t\right)=-i\kappa_{1,3}^{2}\sigma_{ba_{1,2}},$$

where $\kappa_{1,3}^{2}=\frac{N|d_{a_{1,2}b}{|}^{2}\omega_{1,3}}{2\hbar \epsilon_{0}}$. *N* is the density of excitons and $d_{a_{1,3}b}$ are the transition dipole matrix elements of specific transitions, $\omega_{1,3}$ are the probe and signal frequencies and $\epsilon_{0}$ is the vacuum dielectric permittivity. $\sigma_{ij}\left(z,t\right),\;i,j\in \left\{a_{1},a_{2},b,c\right\}$ denotes the density matrix for an exciton at position *z* and time *t*.

$\Omega_{1,3}\left(z,t\right)=\frac{d_{a_{1,3b}}\varepsilon_{1,3}\left(z,t\right)}{\hbar }$ and $\Omega_{2}\left(z,t\right)=\frac{d_{ac}\varepsilon_{2}\left(z,t\right)}{\hbar }$ are the Rabi frequencies of the probe, signal and control fields corresponding to the particular couplings. The key of cross-Kerr nonlinearity lies in the fact that the phase of one light field is modified by an amount determined by the intensity of another optical field. The necessary conditions to achieve a significant cross-phase modulation (over π) are the small absorption and a steep dispersion, which are accomplished due to the EIT. A considerable reduction of the group velocities for both pulses traveling inside the medium allows these two optical fields to mutually interact in a common transparency window for a sufficiently long time. Rebic et al. have shown that by slightly departing from exact resonance conditions, one can obtain a group velocity matching and strong cross-Kerr modulation [34], which facilitates the phase gate operation in this system.

To theoretically describe the setup in the inverted Y configuration, we used the standard method to derive the formula for the susceptibilities, solving the set of stationary Bloch equations in the limit of low probe and signal intensities [35]. The derivation of susceptibility proceeds in a similar way as it was shown in [28]. We expand the system considered in [28] with an additional energy level and after solving the set of Bloch equations in the stationary regime (neglecting nonlinear potential), we obtain the susceptibilities for both probe and signal fields $\Omega_{1}$ and $\Omega_{3}$ in the form [36]:

(2) $$\begin{array}{c} \chi_{1}\left(\delta_{1},\delta_{3},\Omega_{1},\Omega_{3}\right)=\frac{N|d_{ba_{1}}{|}^{2}}{\hbar \varepsilon_{0}}\frac{\sigma_{a_{1}b}}{\Omega_{1}},\\ \chi_{3}\left(\delta_{1},\delta_{3},\Omega_{1},\Omega_{3}\right)=\frac{N|d_{ba_{2}}{|}^{2}}{\hbar \varepsilon_{0}}\frac{\sigma_{a_{2}b}}{\Omega_{3}}. \end{array}$$

Because the probe and signal fields are weaker than the control one $|\Omega_{1}{|}^{2},|\Omega_{3}{|}^{2}<<{\left|\Omega_{2}\right|}^{2}$, the above expressions for susceptibilities can be expanded into Taylor series

(3) $$\begin{array}{c} \chi_{1}\approx \chi_{1}^{\left(1\right)}+\chi_{11}^{\left(3\right)}|\Omega_{1}{|}^{2}+\chi_{13}^{\left(3\right)}{\left|\Omega_{3}\right|}^{2},\\ \chi_{3}\approx \chi_{3}^{\left(1\right)}+\chi_{33}^{\left(3\right)}|\Omega_{3}{|}^{2}+\chi_{31}^{\left(3\right)}{\left|\Omega_{1}\right|}^{2}, \end{array}$$

where $\chi_{1}^{\left(1\right)}$ and $\chi_{3}^{\left(1\right)}$ are the linear part of electric susceptibilities, $\chi_{11}^{\left(3\right)}$ and $\chi_{33}^{\left(3\right)}$ describe self-Kerr phase modulation (i.e., when an optical field modifies its own phase), and $\chi_{13}^{\left(3\right)}$, $\chi_{31}^{\left(3\right)}$ are responsible for cross-phase modulation. The various detunings and relaxation rates present in the system can be grouped in the following notation:

(4) $$\begin{array}{ccc} \Delta_{a_{1}b} & = & -\delta_{1}+i\Gamma_{a_{1}b},\\ \Delta_{cb} & = & -\delta_{2}+i\gamma_{cb},\\ \Delta_{a_{2}b} & = & -\delta_{3}+i\Gamma_{a_{1}b},\\ \Delta_{a_{1}c} & = & -\delta_{1}-\delta_{2}+i\Gamma_{a_{2}b},\\ \Delta_{a_{1}a_{2}} & = & -\delta_{1}-\delta_{3},\\ \Delta_{ca_{2}} & = & -\delta_{3}-\delta_{2}+i\Gamma_{a_{2}b}. \end{array}$$

where the parameters $\Gamma_{ij}$, i≠j describe the damping of exciton states and are determined by temperature-dependent homogeneous broadening due to phonons and broadening due to structural imperfections and eventual impurities. The relaxation damping rates for the coherence are denoted by $\gamma_{ij}\approx \Gamma_{ij}/2$, i≠j [37]. To simplify the expressions describing the susceptibility, we define the following functions of the probe field $\Omega_{1}$:

(5) $$\begin{array}{c} O_{1}=\Omega_{1}+\Delta_{a_{1}b},\\ O_{2}=\Omega_{1}+\Delta_{a_{1}c},\\ O_{3}=\Omega_{1}+\Delta_{a_{2}b}^{*},\\ O_{4}=\Omega_{1}+\Delta_{ca_{2}}. \end{array}$$

After some calculations, we arrive at the linear, self-Kerr, and cross-Kerr parts of susceptibility (Equation (3)) in the following forms:

(6) $$\begin{array}{ccc} \chi_{1}^{\left(1\right)} & = & \frac{\chi_{0}}{O_{1}-\frac{\Omega_{2}^{2}}{O_{2}}},\\ \chi_{11}^{\left(3\right)} & = & \frac{0.5\chi_{0}\Omega_{1}^{2}}{\Delta_{ca_{1}}^{*}}\frac{\chi_{0}}{O_{1}-\frac{\Omega_{2}^{2}}{O_{2}}},\\ \chi_{13}^{\left(3\right)} & = & \chi_{0}\left[\frac{\Delta_{a_{2}b}^{*}}{\Delta_{cb}^{*}}\frac{1}{O_{1}-\frac{\Omega_{2}^{2}}{O_{2}}}\left(\frac{0.5}{O_{1}-\frac{\Omega_{2}^{2}}{O_{2}}}+\frac{0.5}{O_{3}-\frac{\Omega_{2}^{2}}{O_{4}}}\right)-\frac{0.5}{\Delta_{ca_{2}}^{*}}\frac{\Omega_{3}^{2}}{\left(O_{1}-\frac{\Omega_{2}^{2}}{O_{2}}\right)\left(O_{3}-\frac{\Omega_{2}^{2}}{O_{4}}\right)}\right], \end{array}$$

where $\chi_{0}$ is a constant given by [27]

(7) $$\begin{array}{c} \chi_{0}=\frac{N|d_{ba_{i}}{|}^{2}}{\hbar \epsilon_{0}},\;\;\;\;\;\;\left(i=1,2\right). \end{array}$$

In analogy to the procedure presented above, with the following functions of the signal Rabi frequency $\Omega_{3}$

(8) $$\begin{array}{c} O_{1}^{\prime }=\Omega_{3}+\Delta_{a_{2}b},\\ O_{2}^{\prime }=\Omega_{3}+\Delta_{ca_{2}},\\ O_{3}^{\prime }=\Omega_{3}+\Delta_{a_{1}b}^{*},\\ O_{4}^{\prime }=\Omega_{3}+\Delta_{ca_{1}}, \end{array}$$

one arrives at the expressions for the three parts of electric susceptibility for the signal field $\Omega_{3}$

(9) $$\begin{array}{ccc} \chi_{3}^{\left(1\right)} & = & \frac{\chi_{0}}{O_{1}^{\prime }-\frac{\Omega_{2}^{2}}{O_{2}^{\prime }}},\\ \chi_{33}^{\left(3\right)} & = & \frac{0.5\chi_{0}\Omega_{3}^{2}}{\Delta_{ca_{2}}^{*}}\frac{\chi_{0}}{O_{1}^{\prime }-\frac{\Omega_{2}^{2}}{O_{2}^{\prime }}},\\ \chi_{31}^{\left(3\right)} & = & \chi_{0}\left[\frac{\Delta_{a_{1}b}^{*}}{\Delta_{cb}^{*}}\frac{1}{O_{1}^{\prime }-\frac{\Omega_{2}^{2}}{O_{2}^{\prime }}}\left(\frac{0.5}{O_{1}^{\prime }-\frac{\Omega_{2}^{2}}{O_{2}^{\prime }}}+\frac{0.5}{O_{3}^{\prime }-\frac{\Omega_{2}^{2}}{O_{4}^{\prime }}}\right)-\frac{0.5}{\Delta_{ca_{2}}^{*}}\frac{\Omega_{1}^{2}}{\left(O_{1}^{\prime }-\frac{\Omega_{2}^{2}}{O_{2}^{\prime }}\right)\left(O_{3}^{\prime }-\frac{\Omega_{2}^{2}}{O_{4}^{\prime }}\right)}\right]. \end{array}$$

Although formulas for susceptibilities in Equations (6) and (9) have a complex form, their dependence on detunings is visible. The resonance or equal detunings give rise to similar dispersive properties for both 1 and 3 fields, while the nonlinear susceptibility vanishes and the XPM will not occur. Disturbing the EIT conditions by choosing different, but sufficiently small (to still remain in the common transparency window and preserving small absorption) detunings enables one to obtain a nonlinear contribution to susceptibility. The refraction indices $n_{1,3}=\sqrt{\epsilon_{b}+\chi {}_{1,3}}$, where $\epsilon_{b}=7.5$ is the bulk permittivity of Cu_2_O, together with the definition of the group velocity

(10) $$v_{g}=\frac{c}{n_{g}}=\frac{c}{n+\omega \frac{dn}{d\omega }},$$

where $n_{g}$ is the group index, enables one to obtain the expressions for the group velocities of the propagating pulses

(11) $$\begin{array}{c} v_{g}^{\left(1\right)}=\frac{A}{\omega_{1}{\left|d_{a_{1}b}\right|}^{2}}\left(\Omega_{2}^{2}+\Omega_{3}^{2}\right)\\ v_{g}^{\left(3\right)}=\frac{A}{\omega_{3}{\left|d_{a_{2}b}\right|}^{2}}\left(\Omega_{2}^{2}+\Omega_{1}^{2}\right), \end{array}$$

where $A=\frac{4\pi c\varepsilon_{0}}{N}$. From the above equations it follows that, because the orders of dipole moments are almost equal, changing the intensity of the control field and the probe or signal fields, it is possible to match in such a way their group velocities and therefore they can interact mutually in transparent medium for a sufficiently long time. The propagation equations for fields $\Omega_{1}$ and $\Omega_{3}$ have the following form:

$$\begin{array}{c} \left(i\frac{\partial }{\partial z}+\frac{\delta_{1}}{c}-\frac{\Delta \omega_{1}}{c}\right)\Omega_{1}=-\frac{\omega_{1}}{2c}\chi_{1}\Omega_{1},\\ \left(i\frac{\partial }{\partial z}+\frac{\delta_{3}}{c}-\frac{\Delta \omega_{3}}{c}\right)\Omega_{3}=-\frac{\omega_{3}}{2c}\chi_{3}\Omega_{3}, \end{array}$$

where $\Delta \omega_{i}=\omega_{2}-\omega_{i}$. Even inside the transparency window, a realistic medium is characterized by non-zero absorption coefficients

(12) $$\alpha_{1,3}=\frac{\omega_{1,3}}{c}Im\sqrt{1+\chi_{1,3}}.$$

\Assuming that both pulses propagate in the *z*-direction through the sample of length *L*, their amplitudes are constant ($\Omega_{01,3}=\Omega_{1,3}\left(z=0,\delta_{1,3}\right)=const$). The transmissions coefficients for probe and signal fields are defined by the following formulae:

(13) $$\begin{array}{c} T_{1}\left(\delta_{1}\right)=\frac{\Omega_{1}\left(L,\delta_{1}\right)}{\Omega_{10}},\\ T_{3}\left(\delta_{3}\right)=\frac{\Omega_{3}\left(L,\delta_{3}\right)}{\Omega_{30}}. \end{array}$$

The phase difference is equal to the difference of optical path lengths

(14) $$\begin{array}{c} \phi_{1}\left(\omega \right)=\frac{(\omega_{1}n_{1}-\omega_{3}n_{3})L}{c}. \end{array}$$

## 3. Numerical Results

**Figure 2:**
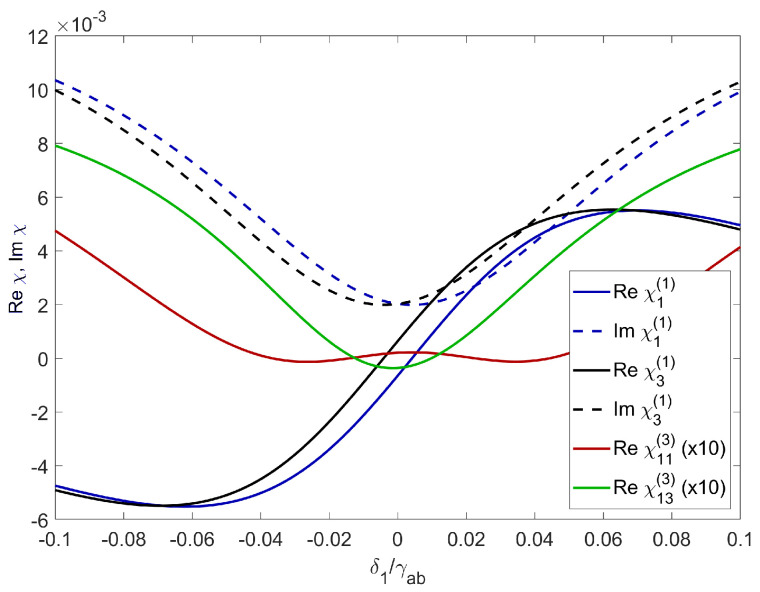
Linear and nonlinear parts of signal field susceptibilities (real and imaginary part) $\chi_{1}^{\left(1\right)}$, $\chi_{3}^{\left(1\right)}$, $\chi_{11}^{\left(3\right)}$, $\chi_{13}^{\left(3\right)}$ for both probe and signal fields. The Rabi frequencies are $\Omega_{2}=600$ GHz, $\Omega_{1}=\Omega_{3}=60$ GHz, exciton density is $N=5.4\times 10^{15}$ cm^−3^.

**Figure 3:**
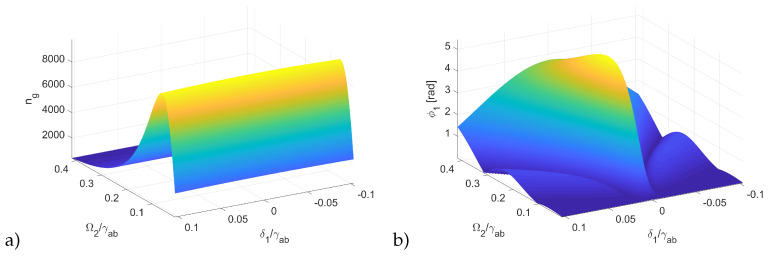
(**a**) Group velocity index and (**b**) cross-phase modulation as a function of detuning $\delta_{1}$ and control field Rabi frequency $\Omega_{2}$, for the same parameters as in Figure 2.

As an example of an excitonic system where XPM can be realized, we used Cu_2_O crystal of thickness L=200 μm. The probe field coupled the ground state *b* (Figure 1) and sublevels of $n_{1}=2$, obtained by Zeeman split of the excitonic state in magnetic field. As a result, we obtained two levels shifted by $\delta_{1}=-\delta_{3}=10$ GHz. The control field coupled the $n_{1}$ state with the empty upper state $n_{2}=10$. The exciton density was $N=5.4\times 10^{15}$ cm^−3^, which was limited by the Rydberg blockade effect caused by the populated lower state $n_{1}$ [27]. In the case of $n_{1}=2$, the upper density limit was $2.6\times 10^{16}$ cm^−3^ [21]. The Rabi frequency of the control field was $\Omega_{2}=600$ GHz, which is comparable to the dissipation rate of the lower state $\gamma_{ab}=2140$ GHz [38] for the temperature, T=10 K. The calculated susceptibility is shown in the Figure 2. One can see that the real parts of both susceptibilities exhibited steep, normal dispersions, while the imaginary parts featured transparency windows in the form of dips. The transparency windows of both probe and signal fields overlapped, providing a common spectral region of small absorption, where the pulses can propagate. Due to the presence of detunings $\delta_{1}$, $\delta_{2}$, there was a noticeable frequency shift between both windows and dispersions, which resulted in a phase difference between propagating signals given by Equation (14). Figure 3a shows the group velocity index $n_{g}$ of the signal field $\Omega_{1}$ inside the transparency window as a function of control field $\Omega_{2}$. One can see that there is an optimum value $\Omega_{2}/\gamma_{ab}\approx 0.15$, for which the slowdown was the strongest. For this strength of the control field, the transparency window was fully formed, but still narrow enough to provide a steep normal dispersion. The obtained slowdown was on the order of $10^{4}$, which means that for the given sample thickness L=200 μm, the propagation time through the crystal was τ∼7 ns. This corresponds to the pulse spectral width Δω∼15 MHz, which is well below the width of the transparency window. Figure 3b shows the calculated cross-phase modulation. The maximum value of $\varphi_{1}∼4.4$ rad was obtained in a wide range of control field strengths, centered around $\Omega_{2}\approx 500$ GHz. As pointed out by Feizpour [39], the phase modulation scales as $1/\Delta_{EIT}$, where $\Delta_{EIT}$ is the spectral width of the window, provided that the window is wide enough and is limited by the strength of the control field $\Omega_{2}$ and dissipation rate $\gamma_{ab}$. In principle, one can use higher excitonic states with smaller $\gamma_{ab}$ to obtain a much narrower transparency window. However, in our calculations this benefit was offset with significantly smaller exciton density. This in turn resulted in a smaller value of susceptibility which produced a smaller phase shift. However, a large real part of susceptibility is accompanied by a significant imaginary part which results in absorption. Due to the particularly large dissipation constants as compared to atomic EIT system, one can observe in Figure 2 that even inside the transparency window Im $\chi ∼10^{-3}$, which resulted in absorption on the order of α∼20 cm^−1^. According to Equation (13), this corresponds to about 70% transmission through a L=200 μm sample. The absorption coefficient is consistent with experimental results by Malerba et al. [40], where the measured values outside the resonance peaks were in the range of $10^{1}$–$10^{2}$ cm^−1^, depending on sample thickness. To sum up, the imaginary part of χ inside the transparency window provided a contribution to absorption that was on the same order as the intrinsic, bulk absorption due to the defects [40] and was sufficiently low to ensure considerable transmission. Since the phase shift was directly proportional to *L*, there was an interplay between XPS and signal transmission.

**Figure 4:**
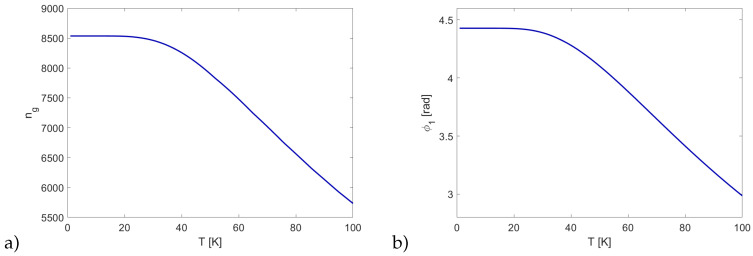
(**a**) Group velocity index and (**b**) cross-phase modulation as a function of temperature.

Finally, we investigated how the XPS scaled with temperature by applying the excitonic line-broadening model described in [27] to the system described above. Figure 4a depicts the maximum slowdown as a function of temperature. For the chosen transparency window width, the slowdown was largely unaffected by broadening up to T∼40 K. Likewise, the cross-phase modulation shown in Figure 4b exhibits identical behavior. This result is consistent with the findings presented in [39], where a similar dependence of XPS on the dephasing rate is shown. Notably, the slowdown and XPS remained significant even at T=100 K. This is possible mainly due to the choice of a low-lying state $n_{1}=2$. As mentioned before, for upper states, the optimal results are obtained with much narrower transparency windows, which are more disturbed by line broadening, which is also much greater for these higher Rydberg states.

We emphasize that all presented numerical results are based on a realistic and experimentally verified parameters for RE in Cu_2_O. We used the usual theoretical approach to derive the formula for the susceptibilities by solving the set of stationary Bloch equations in the low range of probe and signal intensities [36]. The calculated values of cross-phase modulation represent a similar dependence on the control field intensity as those measured recently by Sinclair et al. [41] in a cold Rubidium gas.

## 4. Conclusions

In this paper, we studied the nonlinear response of Rydberg excitons in Cu_2_O sample in an inverted Y configuration, where the incident probe and signal fields interact in EIT conditions. By expanding the ladder system presented in [28] with additional signal field and adjusting the parameters to enter the nonlinear regime, we derived expressions for the third-order susceptibility and suggested the optimal set of parameters for which the remarkable nonlinearities in Cu_2_O with RE might be experimentally realized. Rydberg excitons in Cu_2_O have now reached a stage at which the coherent quantum effects and controlled quantum manipulations could be realized. With Rydberg atoms, it has been possible to obtain a large optical nonlinearity at the single photon level and perform many sophisticated quantum optics experiments such as optical Kerr effect or correlated states [11]. It is expected that the medium of Rydberg excitons is also a fertile area [22,23,32,35,41].

We have demonstrated that it is possible in principle to achieve a phase difference of over π in a 200 μm sample, at temperatures approaching 100 K and exciton densities an order of magnitude below the limit imposed by Rydberg blockade. Since their discovery in 2014, the Rydberg excitons in Cu_2_O have been investigated mostly from the spectroscopic point of view while only a few experiments have focused on their quantum optical applications [22,23], which have confirmed their usefulness in quantum information processing. We hope that our investigations will help in the use of REs as intermediaries in photon–matter coupling in the field of modern quantum processing in solids.
